# Sequential Fractionation of *Opuntia ficus-indica* (L.) Reveals Isorhamnetin Glycosides Associated with Reduced Hepatic Lipid Accumulation and Oxidative Stress

**DOI:** 10.3390/foods15162939

**Published:** 2026-08-21

**Authors:** Jorge Alberto Uribe-Echeverría, Marilena Antunes-Ricardo

**Affiliations:** 1 School of Engineering and Science, Tecnologico de Monterrey, Ave. Eugenio Garza Sada 2501 Sur, Col: Tecnológico, Monterrey 64700, NL, Mexico; 2 Institute for Obesity Research, Tecnologico de Monterrey, Ave. Eugenio Garza Sada 2501 Sur, Col: Tecnológico, Monterrey 64700, NL, Mexico

**Keywords:** flavonoids, functional food, hepatic steatosis, lipid accumulation, metabolic disturbances, sequential exhaustive extraction (SEE)

## Abstract

Metabolic dysfunction-associated fatty liver disease (MAFLD) is the most prevalent chronic liver disease worldwide, affecting 37–52% of the population and potentially progressing to cirrhosis and hepatocellular carcinoma. Effective treatments remain limited, making nutraceuticals such as flavonoids promising therapeutic alternatives. *Opuntia ficus-indica* (L.) (*OFI*) has demonstrated beneficial effects against MAFLD, although its active compounds remain unclear. In this study, the complexity of the OFI sample was addressed through fractionation by sequential exhaustive extraction (SEE), resulting in six fractions enriched with different compound families, and metabolic markers related to steatosis were evaluated. Butanol (BT), ethanol (ET), and water (WA) extracts reduced lipid accumulation by 18–21%. BT and WA fractions also improved glucose uptake and decreased ketone body and reactive oxygen species production. UPLC-MS analysis showed that the WA extract was rich in piscidic acid and exhibited the highest antioxidant activity, whereas the BT displayed the strongest antisteatotic effect. Glycosylated flavonoids, representing 78% of the BT, were the predominant compounds. Isorhamnetin-glucosyl-rhamnosyl-rhamnoside and isorhamnetin-glucosyl-pentoside were inversely associated with triglyceride release, oxidative stress, and ketone body production. These findings identify isorhamnetin derivatives as key bioactive compounds underlying OFI’s beneficial effects against hepatic steatosis.

## 1. Introduction

Chronic diseases associated with nutrition are the leading cause of deaths worldwide and include obesity, diabetes, cardiovascular accidents, Alzheimer’s, and metabolic dysfunction associated with fatty liver (MAFLD) [1]. In a general context, these chronic nutrition diseases share systemic inflammation and metabolic dysregulation triggered by poor nutritional patterns as a core pathophysiological driver [2,3]. Particularly, MAFLD is the most prevalent chronic disease globally; the prevalence in 2021 was approximately 38.0% worldwide and continues to increase [4]. MAFLD is characterized by fat accumulation in the liver of at least 5% of its weight, mainly composed of triglycerides and cholesterol [5]. MAFLD, previously known as non-alcoholic fatty liver disease (NAFLD), is a complex disease that includes environmental factors and preexisting metabolic conditions [6]. Its progression is mediated by the increase in inflammation by the Kupffer cells in the liver, evolving to a metabolically dysfunctional steatohepatitis (MASH). During MASH, the hepatocytes and Kupffer cells produce excessive reactive oxygen species (ROS), which activate stellate cells and, in turn, lead to fibrosis and, ultimately, cirrhosis or hepatocarcinoma, affecting the patient’s health and potentially risking his life [5]. Current treatment consists of diet and exercise, while pharmacological alternatives are limited and exhibit severe side effects [7,8]. In this context, many efforts are underway to develop new strategies to prevent or mitigate MAFLD progression in the long term, such as incorporating functional foods or supplements into the diet.

The incorporation of functional foods into the diet has experienced exponential growth in recent years due to their scientifically proven benefits for nutrition and health. These functional foods contain a varied range of bioactive compounds that can naturally maintain homeostasis and modulate physiological responses to prevent diseases or improve related alterations [9]. *Opuntia ficus-indica* (L.) (OFI) is a widely distributed cactus plant belonging to the Cactaceae family, recognized for its high adaptability to extreme climatic conditions and as a natural source of bioactive compounds attributed with many of the health benefits. Different bioactive molecules have been identified in this plant, including polysaccharides, sterols, phenolic acids, and flavonoids, with isorhamnetin in its various glycosylated forms serving as its chemical fingerprint [10].

Previous studies have demonstrated that supplementation with 4% dried *Opuntia* cladodes in obese Zucker rats (fa/fa) for 7 weeks reduced triglyceride content by 50% [11]. Similarly, *Opuntia* spp. flour supplementation decreased the metabolic abnormalities associated with MAFLD and modified the gut microbiota in obese rats [12,13]. Likewise, diet supplementation with hydroalcoholic extracts obtained from *Opuntia* spp. fruit (rich in betalains) exhibited beneficial effects on inflammation, oxidative stress, lipid metabolism, and the gut microbiota in steatotic-induced in vivo models [14,15,16].

Other studies have demonstrated that extracts rich in phenolic acids and flavonoids mitigate the metabolic alterations associated with MAFLD by reducing acetyl carboxylase and fatty acid synthase through AMPK activation [17]. Likewise, the protective and beneficial effects of individual phytochemicals against different MAFLD alterations have been evaluated. Pterostilbene, a polyphenolic compound belonging to the stilbene family, has been shown to reduce steatosis in a high-fat diet animal model by inducing changes in riboflavin and nicotinamide mononucleotide metabolism [18].

Rhein, an anthraquinone metabolite, has shown protective effects against HFD-induced MAFLD by activating the 5′-adenosine monophosphate-activated protein kinase (AMPK)/acetyl-CoA carboxylase (ACC)/sterol regulatory element-binding protein 1 (SREBP1) signaling pathway, reducing steatosis, and alleviating liver injury and inflammation [19]. Phenolic acids, such as gallic and ellagic acids, exhibit protective effects against oxidative stress, inflammation, and metabolic alterations associated with MAFLD by modulating the IRF6/PPARγ signaling pathway and lipid/amino acid metabolism [20,21,22,23]. Likewise, the flavonoid genistein ameliorates glucose–lipid metabolism and liver function disorders associated with MAFLD and improves intestinal barrier integrity, alleviating hepatic inflammation [24].

Even though plant-based extracts have shown promising functional food properties due to their bioactive phytochemical content, they have two significant limitations: the complexity of the matrix and variability between harvests [25]. For example, *Opuntia ficus-indica* (L.) contains different compounds that coexist in the plant. The phytochemical profile will depend not only on the plant’s growth conditions (temperature, humidity, and nutrient availability) and biotic stressors, but also on the extraction method used to obtain its bioactive compounds [26,27,28]. These changes in the phytochemical profile increase complexity and delay understanding of the biochemical mechanisms involved, limiting the scalability and scope of research aimed at designing personalized foods.

An easy, reproducible, and cost-effective strategy to address this limitation is serial exhaustive extraction (SEE), which groups phytochemical compounds from a complex matrix into different extracts based on their structural or polarity characteristics [29]. Using SEE, it is possible to extract a wide range of polyphenols with different polarities, which otherwise could not be recovered due to their diverse structures and physicochemical properties [30]. Likewise, this technique significantly increases the overall polyphenol extraction yield [31]. Through this strategy, it will be possible to associate the contribution of compound families in modulating or mitigating the metabolic alterations associated with MAFLD. Taking everything into account, this work aimed to evaluate the relationship between the chemical profiles of different extracts of *Opuntia ficus-indica* (L.) cladode flour obtained via serial exhaustive extraction (SEE) and the specific contributions to their effects on metabolic disturbances associated with MAFLD using an in vitro hepatic steatosis model.

## 2. Materials and Methods

### 2.1. Chemicals and Reagents

For the extractions, *n*-hexane was purchased from Fermont (Monterrey, NL, Mexico), petroleum ether from DEQ (Monterrey, NL, Mexico), and ethyl acetate from Honeywell (Charlotte, NC, USA). *n*-Butanol was purchased from DEQ (Monterrey, NL, Mexico), 96% ethyl alcohol from AZ (Guadalajara, JA, Mexico), and ultrapure water from a Milli-Q Integral 15 system (Darmstadt, HE, Germany). For chromatographic analysis, mass-grade acetonitrile was purchased from TEDIA (Fairfield, OH, USA), mass-grade water from Honeywell (Charlotte, NC, USA), and Formic acid from Sigma-Aldrich (St. Louis, MO, USA).

### 2.2. Plant Material

*Opuntia ficus-indica* (L.) plants were grown in Montemorelos, Nuevo León, Mexico, and were processed to obtain the flour as described previously [32]. Briefly, the cladodes were sanitized with sodium hypochlorite (200 ppm) for 10 min. Then, the cladodes were dehydrated in a convection oven for 8 h at 60 °C. Finally, the cladodes were ground in a hammer mill (PULVEX 200, Mexico City, Mexico) using a 2 mm round-orifice screen. The *Opuntia ficus-indica* (L.) flour used in this study was donated by Alimentos Funcionales S. de R.L. M.I.

#### Proximate Composition Analysis

The proximate composition of *Opuntia ficus-indica* cladodes was determined according to the official methods of the Association of Official Analytical Chemists (AOAC International) as described in [33]. Moisture (AOAC 925.10), ash (AOAC Method 923.03), crude protein (Kjeldahl method; AOAC Method 978.02), crude fat (Soxhlet extraction; AOAC Method 920.85), and total dietary fiber (enzymatic–gravimetric method; AOAC Method 991.43). Available carbohydrates were calculated by difference. All analyses were performed in triplicate, and the results are expressed as mean ± standard deviation on a fresh weight basis.

### 2.3. Opuntia ficus-indica (L.) Extracts Preparation

#### 2.3.1. Sequential Exhaustive Extraction (SEE)

The sequential exhaustive extraction was carried out using a modified method [34] by mixing *Opuntia ficus-indica* (L.) flour with *n*-hexane (HX) at a 1:10 *w*/*v* ratio for 30 min at 30 °C and 200 rpm in an orbital shaker (Orbital MaxQ 4000, Thermo Scientific, Waltham, MA, USA). Afterward, the samples were centrifuged for 10 min at 5000× *g*. Then, the supernatants were collected, and the pellets were used to repeat the process sequentially with solvents of higher polarity: petroleum ether (PE), ethyl acetate (EA), *n*-butanol (BT), 80% ethanol (ET), and water (WA). All the supernatants were collected, and the solvents were removed by evaporation with a Rotavapor R-100 (BÜCHI, Flawil, Switzerland) under the following conditions: 50 mBar, 38 °C, and a velocity of 80 rpm. Then, they were resuspended in water before being frozen at −80 °C. They were then lyophilized (Freezone 12, LABCONCO, Kansas City, MO, USA) at 0.050 mBar. The resulting lyophilized extracts were stored at −80 °C.

#### 2.3.2. Whole Ethanolic Extract (WEE)

As a comparative control of the previously reported literature [14,35], a whole ethanolic extract (WEE) was prepared as follows: *Opuntia ficus-indica* (L.) flour was mixed with ethanol/water (80/20, *v*/*v*) in a 1:10 *w*/*v* ratio and agitated at 250 rpm for 2 h at 35 °C in an orbital shaker (Orbital MaxQ 4000, Thermo Scientific, Waltham, MA, USA). After a 10 min rest, the suspension was filtered through filter paper No. 1 (WhatmanTM, Cytiva, Marlborough, MA, USA). Then, the extract was evaporated using a Rotavapor R-100 (BUCHI, Flawil, Switzerland) under the following conditions: 50 mbar, 38 °C, and 80 rpm. Finally, the extract was frozen at −80 °C before lyophilization (Freezone 12, LABCONCO, Kansas City, MO, USA) at 0.050 mBar. The resulting lyophilized extracts were stored at −80 °C.

### 2.4. In Vitro Hepatic Steatosis Cell Model

#### 2.4.1. Cell Culture

Human hepatocellular (HepG2: HB-8065) cells were acquired from American Type Culture Collection (ATCC) (Manassas, VA, USA). Cells were grown in Dulbecco’s Modified Eagle Medium-F12 (DMEM-F12, Gibco, New York, NY, USA) supplemented with 5% fetal bovine serum (FBS, Corning, New York, NY, USA) and incubated at 37 °C and 5% CO_2_.

#### 2.4.2. Dose Determination by Cell Viability Evaluation with MTT Assay

The viability of HepG2 cells was evaluated using a 3-(4,5-dimethylthiazol-2-yl)-2,5-diphenyltetrazolium bromide (MTT, Sigma-Aldrich, St. Louis, MO, USA) assay. For 96-well plate experiments, 1 × 10^4^ cells per well were seeded. After 24 h, the cells were treated with different concentrations (3.125–200 µg/mL) of WEE (as the reference extract) for 24 and 48 h. Then, the cell viability was determined by adding 100 µL of 3-(4,5-dimethylthiazol-2-yl)-2,5-diphenyltetrazolium bromide (MTT) at 1 mg/mL and incubating for 2 h, and finally eluting the formazan crystals with dimethyl sulfoxide (DMSO, Fermont, Monterrey, NL, Mexico) and reading at 540 nm in a plate reader (Synergy^TM^, Biotek, Winooski, VT, USA). Cell viability was expressed as a percentage relative to the control (non-treated) cells.

#### 2.4.3. Development of Oleic Acid (OA)-Induced Hepatic Steatosis Model in HepG2 Cells

Oleic acid (OA)-Bovine Serum Albumin (BSA) solution: oleic acid was prepared by making 10 mL of an 11% (*w*/*v*) solution of BSA (B2064, Sigma-Aldrich, St. Louis, MO, USA) in water and then adding 38 µL of OA (O1008, Sigma-Aldrich, St. Louis, MO, USA) and mixing. The solution was then rested for 30 min at room temperature and filtered through a 0.22 μm filter.

Lipid accumulation was evaluated in a 96-well plate by seeding 1 × 10^4^ cells per well. After 24 h, the cells were treated with oleic acid (OA) at different concentrations (100, 200, 300 μM) for 24 and 48 h. The cells were washed twice with PBS and fixed with 4% paraformaldehyde (Sigma-Aldrich, St. Louis, MO, USA). Then, the cells were washed with PBS for 5 min and subsequently washed for 15 s with ice-cold 60% isopropanol. Afterward, Oil Red O (ORO) staining was added and incubated at room temperature for 20 min. Then, the wells were washed twice with PBS, and the lipid droplets were eluted with 100% isopropanol by shaking the plate for 30 min at room temperature. Finally, the plate was read at 510 nm in a plate reader (SynergyTM, Biotek, Winooski, VT, USA). Lipid accumulation was expressed as a percentage relative to the non-treated cells.

ORO solution: The ORO stock was prepared by resuspending 250 mg of ORO oil-red O (ORO, Seelze, NI, Germany) powder in 40 mL of isopropanol for 24 h at 60 °C. Then, the ORO working solution was prepared by diluting to 60% with water, then filtered through filter paper No. 1 (WhatmanTM, Cytiva, Marlborough, MA, USA), and subsequently through a 0.22 μm filter.

#### 2.4.4. Effect of *Opuntia ficus-indica* (L.) (OFI) Extracts on Lipid Accumulation in an In Vitro Hepatic Steatosis Model

Human hepatocellular (HepG2) cells were seeded in a 96-well plate at 1 × 10^4^ cells per well and incubated for 24 h at 37 °C and 5% CO_2_. After that, cells were treated with 300 µM OA for another 24 h, followed by each OFI extract at 100 µg/mL for 24 h. Finally, cell viability and lipid accumulation were determined as described above.

#### 2.4.5. Effect of *Opuntia ficus-indica* (L.) (OFI) Extracts on Triglycerides, Glycerol, and Glucose Levels in an In Vitro Hepatic Steatosis Model

The concentrations of triglycerides, glycerol, and glucose were determined in the extracellular medium of oleic acid-induced steatotic HepG2 cells using commercial kits according to the manufacturer’s instructions. The triglyceride concentration was determined as described in the Triglyceride Assay Kit Manual (AB65336, Abcam, Cambridge, UK). The glycerol concentration was determined as described in the Glycerol Assay Kit Manual (AB133130, Abcam, Cambridge, UK). Lastly, glucose concentration was determined using a Glucose (GO) Assay Kit (GAGO20, Sigma-Aldrich, St. Louis, MO, USA). All the assesments were determined using a plate reader (SynergyTM, Biotek, Winooski, VT, USA).

### 2.5. Ketone Bodies Release Determination

The acetoacetate and acetone were determined using a variation of the Imbert method [36] using a reactive solution of 1% sodium nitroprusside (Sigma-Aldrich, St. Louis, MO, USA) with 5% glycine (Sigma-Aldrich, St. Louis, MO, USA). The samples were tested directly by adding 50 μL of the supernatant to 150 µL of the reactive solution and incubating for only 25 min at room temperature, protected from light. The plate was read at 540 nm in a plate reader (SynergyTM, Biotek, Winooski, VT, USA). Ketone body production by treated cells was expressed as a percentage of that in non-treated cells.

### 2.6. Effect of Opuntia ficus-indica (L.) (OFI) Extracts on Modulation of Intracellular Production of Reactive Oxygen Species (ROS) in an In Vitro Hepatic Steatosis Model

The cellular antioxidant activity was determined as reported in the literature with slight modifications [37]. HepG2 cells were seeded at 1 × 10^4^ per well in a 96-well plate, and after 24 h, 300 µM of OA was added to induce lipid accumulation. Then, after 24 h, the cells were treated with OFI extracts at 100 µg/mL and 60 µM of 2′,7′-dichlorofluorescein diacetate (DCFH-DA).

The next day, the cells were washed twice with PBS to remove any excess DCFH-DA, then challenged with an oxidant stimulus using 2,2′-azobis(2-amidinopropane) dihydrochloride (AAPH) at 500 µM. The intracellular ROS production was monitored by measuring fluorescence (Ex/Em: 485/528) every 2 min for 2 h. ROS production in treated cells was expressed as a percentage relative to that in non-treated cells.

### 2.7. Identification and Quantification of Phenolic Compounds Using UPLC-PDA-QDa

Chromatographic analyses were performed in an ultra-performance liquid chromatography system (Waters, Milford, MA, USA) coupled with a photodiode-array (PDA) detector and a Quadrupole Dalton (QDa) Mass Detector. The PDA detector measured the UV-Vis range from 200 to 400 nm; meanwhile, the QDa was operated in positive mode with a capillary voltage of 0.8 kV and a cone voltage of 0.3 kV, detecting masses ranging from 150 to 800 kDa. Separation was performed using a reverse-phase C18 column (Biphenyl^®^ 2.6 μm, 2.1 × 100 mm, Phenomenex, Torrance, CA, USA) at 30 °C. The samples were resuspended in 50% MS-grade methanol and filtered through a 0.22 μm filter. The injection volume was 5 μL, and the separation was completed in 40 min with a flow rate of 0.2 mL/min. Mobile phases consisted of water with 0.1% formic acid (*v*/*v*) (A) and acetonitrile with 0.1% formic acid (*v*/*v*) (B). The chromatographic separation started with 2% B and continued with the following gradient: 1 min, 2% (B); 3 min, 5% (B); 24 min, 35% (B); 28 min, 100% (B); 31 min, 100% (B); and 35 min, 2% (B). The chromatograms were obtained at 280, 320, and 365 nm. Quantification was expressed in μg equivalents (eq) of gallic acid/g OFI extract (y = 41112.18x − 2061.25, R^2^ = 0.9999), μg equivalents (eq) of ferulic acid/g OFI extract (y = 192274.82x − 3707.55, R^2^ = 0.9996), and μg equivalents (eq) of quercetin/g OFI extract (y = 36442.26x + 4969.28, R^2^ = 0.9904) for compounds detected at 280 nm, 320, and 365 nm, respectively.

### 2.8. Statistical Analysis

All experiments were performed in independent replicates, and results were expressed as mean ± standard deviation. Results were analyzed using JPM Pro 14 software (Cary, NC, USA). The comparison between treatments was analyzed using a one-way ANOVA followed by the Tukey post hoc test at a 95% confidence level (*p* < 0.05).

## 3. Results and Discussion

### 3.1. Extraction Yield

Compared with direct hydroalcoholic extraction (WEE), the SEE strategy achieved an extraction yield of 25.6%, whereas hydroalcoholic extraction yielded only 9.04% (Table 1). The greater recovery of total *Opuntia ficus-indica* (L.) extract was obtained using sequential exhaustive extraction (256.16 mg/g of OFI flour). This result agrees with that reported by other authors who found that sequential extraction allows the recovery of 70% more flavonoids than conventional 80% ethanol extraction, since the different polarities of the solvents increase the permeability of plant cells [38].

The extraction yield of whole ethanolic extract (WEE) using 80% ethanol was 1.70-fold higher (99.86 mg/g of OFI flour) than its equivalent extract in the SEE (58.85 mg/g of OFI flour). Among the SEE extracts, *n*-butanol, 80% ethanol, and water extracts account for 91% of the total extract recovery, indicating that most of the recovered bioactive compounds are polar or moderately polar. These results were expected, as the proximate composition of *Opuntia ficus-indica* (L.) cladodes indicates that carbohydrates account for about 15.22% of this food matrix. In contrast, the minority components are lipids, which represent only 3.18% (Table S1).

### 3.2. Induction of the In Vitro Model of Hepatic Steatosis

**Figure 1:**
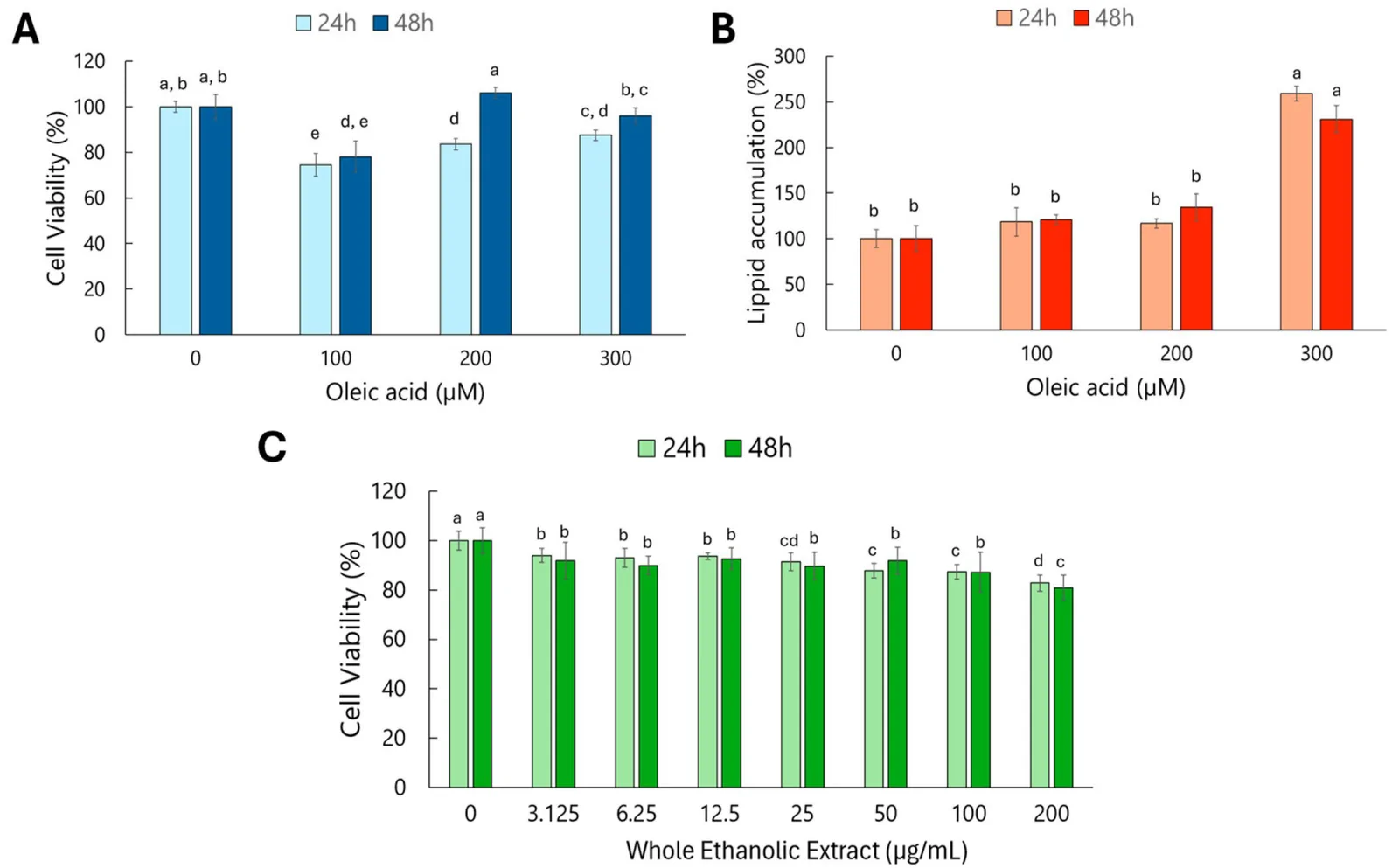
Development of an in vitro model of oleic acid (OA)-induced hepatic steatosis and determination of treatment dose for the extracts using human hepatocellular (HepG2) cells. The cells were treated with different concentrations of oleic acid (100, 200, and 300 µM) for 24 and 48 h. (**A**) Cell viability was assessed with an MTT assay, and (**B**) the lipid accumulation was assessed by Oil Red O (ORO) staining. (**C**) Effect of WEEs on cell viability and oleic acid (OA)-induced hepatic steatosis in an in vitro model using human hepatocellular (HepG2) cells. The cells were treated with different concentrations of the WEE for 24 and 48 h. The results are presented as mean ± standard deviation of three different experiments. ^a–e^ Different letters correspond to significant differences between treatments determined by ANOVA analysis followed by the Tukey test (*p* < 0.05). WEE: whole ethanolic extract.

The highest lipid accumulation in human hepatocellular (HepG2) cells was induced when cells were treated with 300 µM oleic acid (OA) without affecting their viability. Cell lipid accumulation increased by up to 200% (Figure 1A) without affecting cell viability, which remained above 85% at 48 h (Figure 1B). These results are consistent with previous studies that reported a similar increase in lipid accumulation in HepG2 cells (167–300%) using oleic acid concentrations higher than those used in this study [43,44].

### 3.3. Effect of Opuntia ficus-indica (L.) (OFI) Extracts on Lipid Accumulation in an In Vitro Model of Hepatic Steatosis

#### 3.3.1. Cell Viability

There was no significant difference in cell viability at any of the evaluated concentrations of whole ethanolic extract (WEE) at 48 h (Figure 1C). In cells treated with the lowest WEE concentration (3.125 µg/mL), viability was 90%, with no significant change even at elevated concentrations such as 100 µg/mL. Increasing the concentration to 200 µg/mL reduced cell viability to 82%. Therefore, the 100 µg/mL concentration was used in the subsequent experiments.

**Figure 2:**
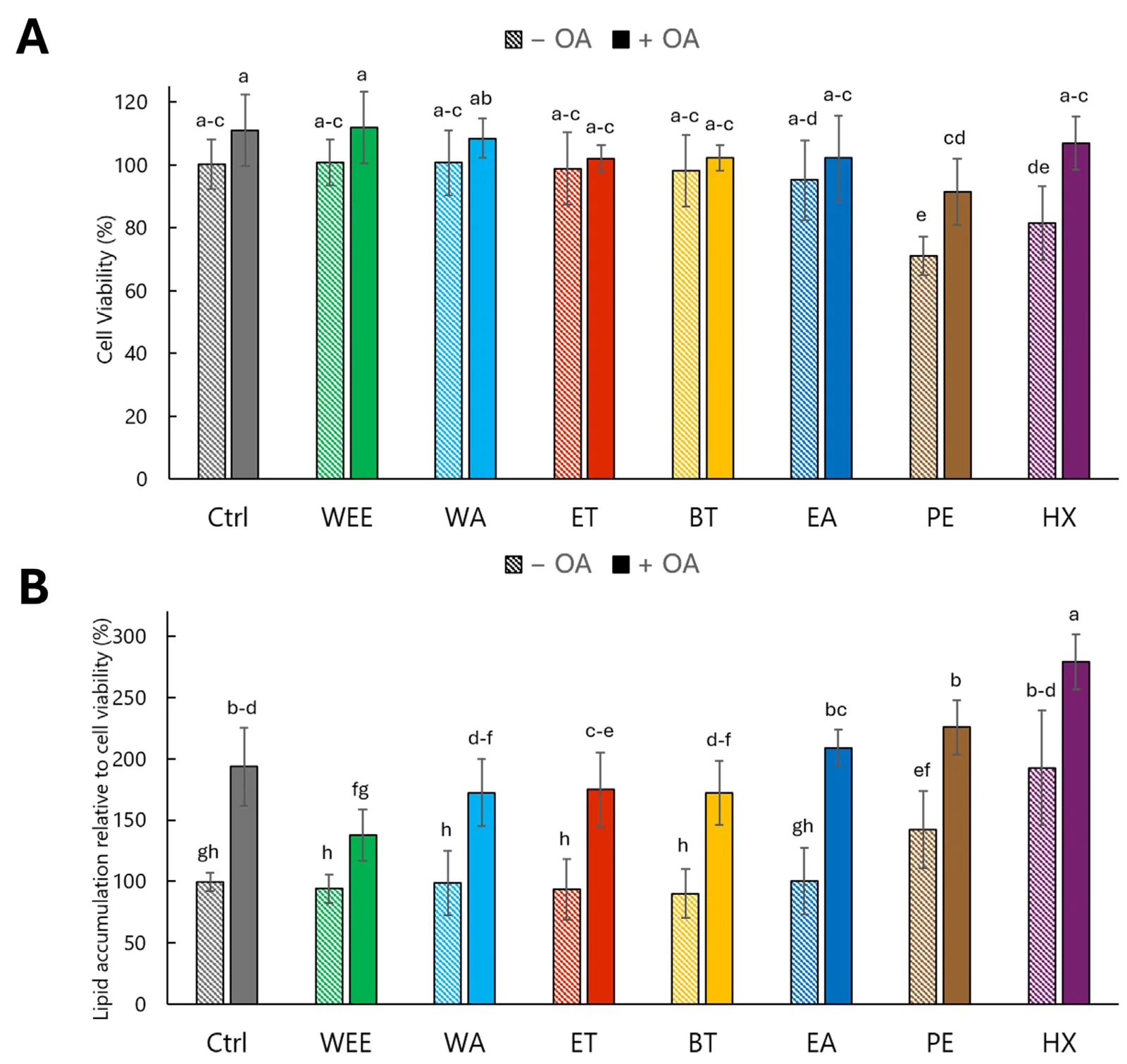
Effect of *Opuntia ficus-indica* (L.) (OFI) extracts on cell viability and lipid accumulation in an in vitro model of oleic acid (OA)-induced hepatic steatosis. Human hepatocellular (HepG2) cells were treated with 100 µg/mL of each *Opuntia ficus-indica* (L.) (OFI) extract with and without the presence of oleic acid (OA). (**A**) Cell viability of HepG2 cells assessed with the MTT assay. (**B**) Lipid accumulation of HepG2 cells determined by Oil Red O (ORO) staining. ^a–h^ Different letters correspond to significant differences between treatments determined by the ANOVA test followed by the Tukey test (*p* < 0.05). OA: oleic acid, WEE: whole ethanolic extract, WA: water, ET: 80% ethanol, BT: *n*-butanol, EA: ethyl acetate, PE: petroleum ether, HX: *n*-hexane.

Regarding the OFI extracts obtained by SEE, *n*-hexane (HX) and petroleum ether (PE) extracts decreased cell viability by 22% and 30%, respectively. (Figure 2A). This behavior could be explained by the extraction of non-polar compounds, such as fatty acids, in these extracts. It is reported that fresh OFI cladode contains 1.15% lipids, with about 24% saturated fatty acids (SFAs), mainly palmitic acid, and 57% unsaturated fatty acids (UFAs), with oleic and linoleic acids as the most abundant [45]. Palmitic acid induces lipotoxicity in hepatocytes through diverse effects, including the upregulation of Kruppel-like factor 7 (KLF7), which increases pro-inflammatory cytokine production and enhances death receptor signaling [46].

Additionally, it increases reactive oxygen species (ROS) production, damages mitochondria, and potentially leads to lysosomal permeabilization, endoplasmic reticulum stress, and cytotoxic effects [47]. This cytotoxic effect observed in the *n*-hexane and petroleum ether extracts was significantly reduced when the cells were treated with oleic acid, indicating a protective effect of oleic acid against the harmful effects of the SFA present in the HX and PE extracts [48,49].

#### 3.3.2. Lipid Accumulation

The *n*-hexane (HX) and petroleum ether (PE) extracts increased the lipid accumulation in cells without OA by 92.5% and 42.2%, respectively, whereas the rest of the extracts did not show changes in lipid accumulation (Figure 2B). In the oleic acid (OA)-induced hepatic steatosis model, non-treated cells increased their lipid content by 93.7%. HX extract increased cell lipid accumulation by almost 85% compared with the control + OA. Due to increased lipid accumulation and reduced viability, the HX and PE extracts were discarded for further analysis.

On the other hand, *n*-butanol (BT), water (WA), and 80% ethanol (ET) extracts showed a slight tendency to reduce intracellular lipid accumulation by 21.65%, 21.29%, and 18.9%, respectively; although this decrease did not reach significance compared to the control + OA. The whole ethanolic extract (WEE), used as the reference extract, showed a significant reduction in lipid accumulation (56.2%) compared with non-treated cells (Ctrl) + OA. BT, WA, and ET extracts were selected as the best candidates for the next experimental assays, with WEE as the reference extract.

### 3.4. Effect of Opuntia ficus-indica (L.) (OFI) Extracts on Triglycerides, Glycerol, and Glucose Levels and Ketone Production in an In Vitro Hepatic Steatosis Model

#### 3.4.1. Triglycerides Release

**Figure 3:**
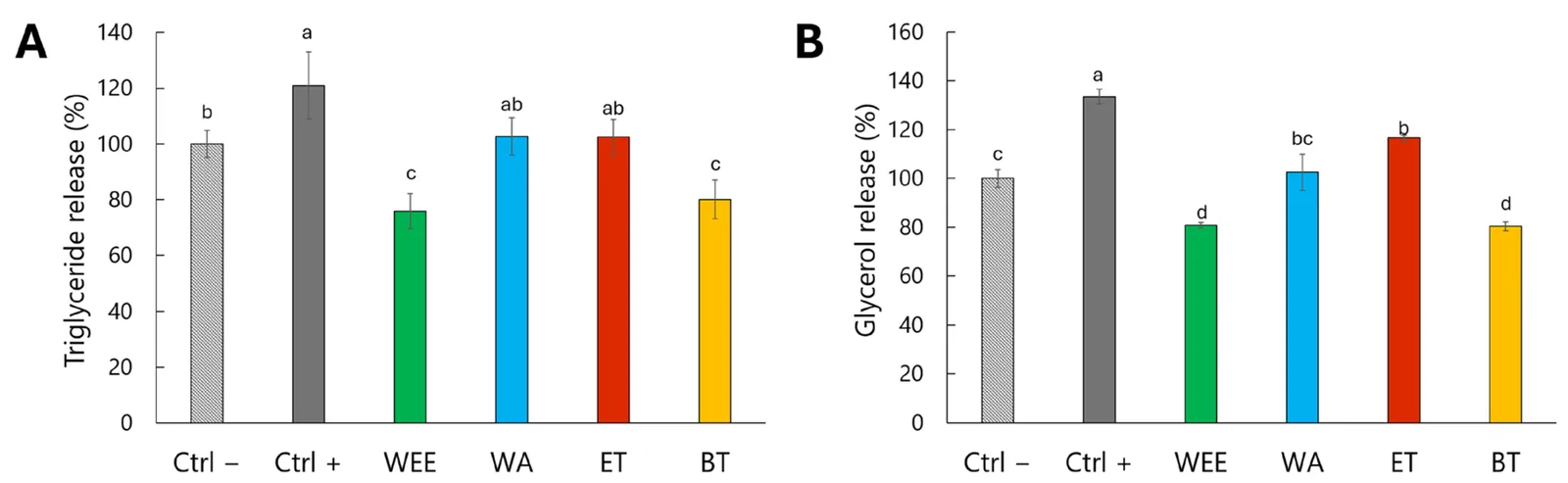
Effect of *Opuntia ficus-indica* (L.) (OFI) extracts on triglyceride (**A**) and glycerol (**B**) release in an oleic acid (OA)-induced hepatic steatosis in vitro model. Human hepatocellular (HepG2) cells were pre-treated with 300 µM OA to induce steatosis, and 24 h later were treated with 100 µg/mL each of OFI extract for another 24 h. The results are presented as mean ± standard deviation of three different experiments. ^a–d^ Different letters indicate significant differences between treatments determined by ANOVA analysis, followed by the Tukey test (*p* < 0.05). Ctrl−: non-treated cells without OA, Ctrl+: non-treated cells + OA, WEE: whole ethanolic extract + OA, WA: water + OA, ET: 80% ethanol + OA, BT: *n*-butanol + OA.

Triglyceride release was determined as an indicator of an active metabolic state of hepatic (HepG2) cells in response to lipid overload. Triglycerides released from non-treated steatotic cells (Ctrl+) increased by 20% compared with non-steatotic cells (Ctrl−), indicating disturbances in lipid metabolism and transport, characteristics of hepatic steatosis (Figure 3A). After treating OA-induced steatotic cells with water (WA) or 80% ethanol (ET) extracts, triglyceride release levels appear to be restored, reaching levels similar to Ctrl− and suggesting an adaptive response of the hepatocyte [50]. On the other hand, cells treated with *n*-butanol (BT), showing an effect similar to that of WEE under steatotic conditions induced by oleic acid (OA), showed a significant reduction in triglyceride release levels compared with both Ctrl− and Ctrl+, suggesting the activation of a different lipid metabolism pathway. A potential alternative hypothesized is the release of very low-density lipoproteins (VLDL), which is the primary source of triglyceride release from hepatocytes [51].

#### 3.4.2. Glycerol Release

The glycerol release levels were 33.44% higher in non-treated steatotic cells (Ctrl+) compared with non-steatotic cells (Ctrl−), indicating the activation of the lipolysis mechanism by the cell in response to lipid overload (Figure 3B). None of the OFI extracts evaluated showed a decrease in glycerol release. In contrast, all treatments showed a significant reduction in these levels, even below the levels in the negative control (Ctrl−). In particular, BTs showed a similar reduction (53.02%) in levels as WEE.

Lipolysis involves the breakdown of triglycerides in hepatic lipid droplets, releasing fatty acids and glycerol [52]. The decrease in glycerol release levels could be related to its use by other metabolic pathways to synthesize molecules [53]. This activation may be linked to the fact that oleic acid (OA) is an even-numbered fatty acid, meaning that its oxidation results in only acetyl-CoA molecules, which can be used for the synthesis of fatty acids, cholesterol, or ketone bodies, but not directly for other molecules such as glucose or pyruvate [54]. The hypothesis is that reductions in glycerol release in treated cells are linked to the cell’s reutilization of glycerol rather than to reduced lipolysis due to reduced lipid accumulation.

#### 3.4.3. Glucose Uptake and Ketone Bodies Release

The liver is the metabolic center of the body because many physiological processes, including the synthesis and degradation of macromolecules such as lipids, carbohydrates, glycogen, and ketone bodies, occur there [55]. The key nodes, Acetyl-CoA and pyruvate, interconnect all these metabolic routes. In this context, other routes may be affected and contribute to reducing lipid accumulation, rather than the release of triglycerides, such as the production of ketone bodies.

**Figure 4:**
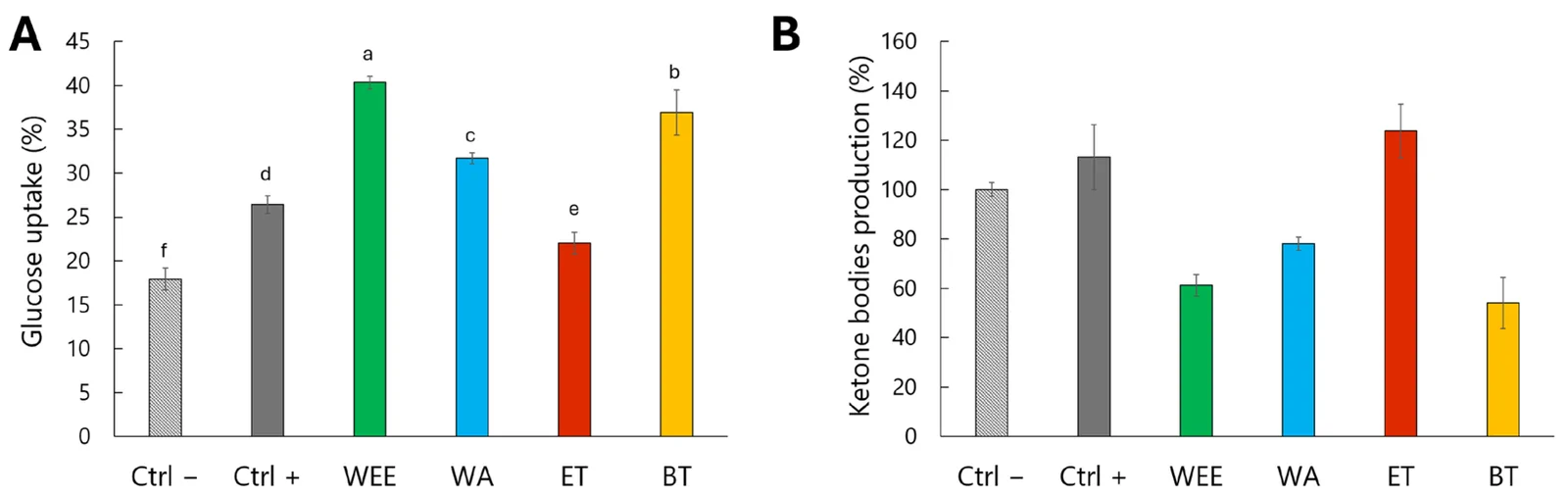
Effect of *Opuntia ficus-indica* (L.) (OFI) extracts on glucose release (**A**) and ketone bodies release (**B**) in an oleic acid (OA)-induced hepatic steatosis in vitro model. Human hepatocellular (HepG2) cells were pre-treated with 300 µM OA to induce steatosis, then treated with 100 µg/mL each of the OFI extracts for an additional 24 h. The results are presented as mean ± standard deviation of three different experiments. ^a–f^ Different letters correspond to significant differences between treatments determined by ANOVA analysis, followed by the Tukey test (*p* < 0.05). Ctrl−: non-treated cells without OA, Ctrl+: non-treated cells + OA, WEE: whole ethanolic extract + OA, WA: water + OA, ET: 80% ethanol + OA, BT: *n*-butanol + OA.

Glucose uptake in non-treated steatotic HepG2 cells (Ctrl+) was significantly higher (~1.5-times) than glucose uptake levels in non-treated cells (Ctrl−) (Figure 4A). It is reported that oleic acid can increase GLUT2 receptor expression in hepatocytes, potentially explaining the increase in glucose uptake [56]. Another possibility is that hepatocytes use glucose for lipid storage, likely as a precursor of glycerol for triglyceride synthesis [57]. Overall, increased glucose uptake in steatotic conditions could be interpreted as a compensatory mechanism that sustains glycolytic flux, glycerol-3-phosphate availability, and nicotinamide adenine dinucleotide phosphate (NADPH) production, thereby maintaining energetic balance and cell survival under lipotoxic stress [58,59]. Further analysis is required to elucidate which specific mechanism is involved.

In this study, results showed that cells treated with water (WA) and *n*-butanol (BT) exhibited significant increases of 5.25% and 10.50%, respectively, in glucose uptake compared to non-treated steatotic cells (Ctrl+), similar to the effect of WEE (13.92%), especially with the BTs. In contrast, cells treated with 80% ethanol (ET) extract exhibited a significant reduction (22%) in glucose uptake with respect to the non-treated steatotic cells (Ctrl+). On the other hand, no significant differences were observed in the ketone body release between non-treated cells (Ctrl−) and non-treated steatotic cells (Ctrl+). But a significant reduction in ketone body release was observed in cells treated with WA (25%) and BT (67%), compared with non-treated steatotic cells (Ctrl+), again showing a very similar behavior to the reference WEE (52%) (Figure 4B). Also, the 80% ethanol (ET) extract increased ketone body release by 10% compared to Ctrl+, but this increase was not statistically significant. These findings suggest that under these treatments, glucose is predominantly used in glycolysis, promoting the Krebs cycle oxidation and limiting its diversion to ketogenesis [60].

The *n*-butanol extract (BT) showed a similar biological behavior to the reference extract (WEE), as the reduction in lipid accumulation observed in steatotic cells treated with these extracts was not accompanied by a decrease in glucose uptake, suggesting a compensatory and efficient metabolic state in which the cell prioritizes energetic homeostasis and lipid management.

The increase in lipolysis and the subsequent fatty acid oxidation is hypothesized to increase the consumption of Krebs cycle intermediates by cataplerotic reactions [56], potentially leading to the activation of anaplerotic pathways to replace these molecules, and pyruvate could be diverted to anaplerotic pathways rather than energy metabolism [61,62]. Likewise, the decrease in lipid accumulation, accompanied by a reduction in ketone body release, as shown in steatotic cells treated with WEE or BT, is an interesting feature that could be desirable during MAFLD treatment by potentially reducing the probability of developing ketoacidosis, one of the most serious complications in patients with diabetes [63]. These results suggest that BTs recovered most of the compounds responsible for the antisteatotic effects observed with WEE treatment.

### 3.5. Effect of Opuntia ficus-indica (L.) (OFI) Extracts on the Intracellular Production of Reactive Oxygen Species (ROS) in an In Vitro Model of Hepatic Steatosis

In the MAFLD context, the presence of ROS is a result of the β-oxidation of fatty acids in mitochondria and peroxisomes; however, the reduction in ROS production observed in non-treated steatotic cells (Ctrl+) compared to control negative cells is associated with the oxidative protective effect derived from the double bond present in unsaturated fatty acids by the direct scavenging of ROS [64,65,66].

**Figure 5:**
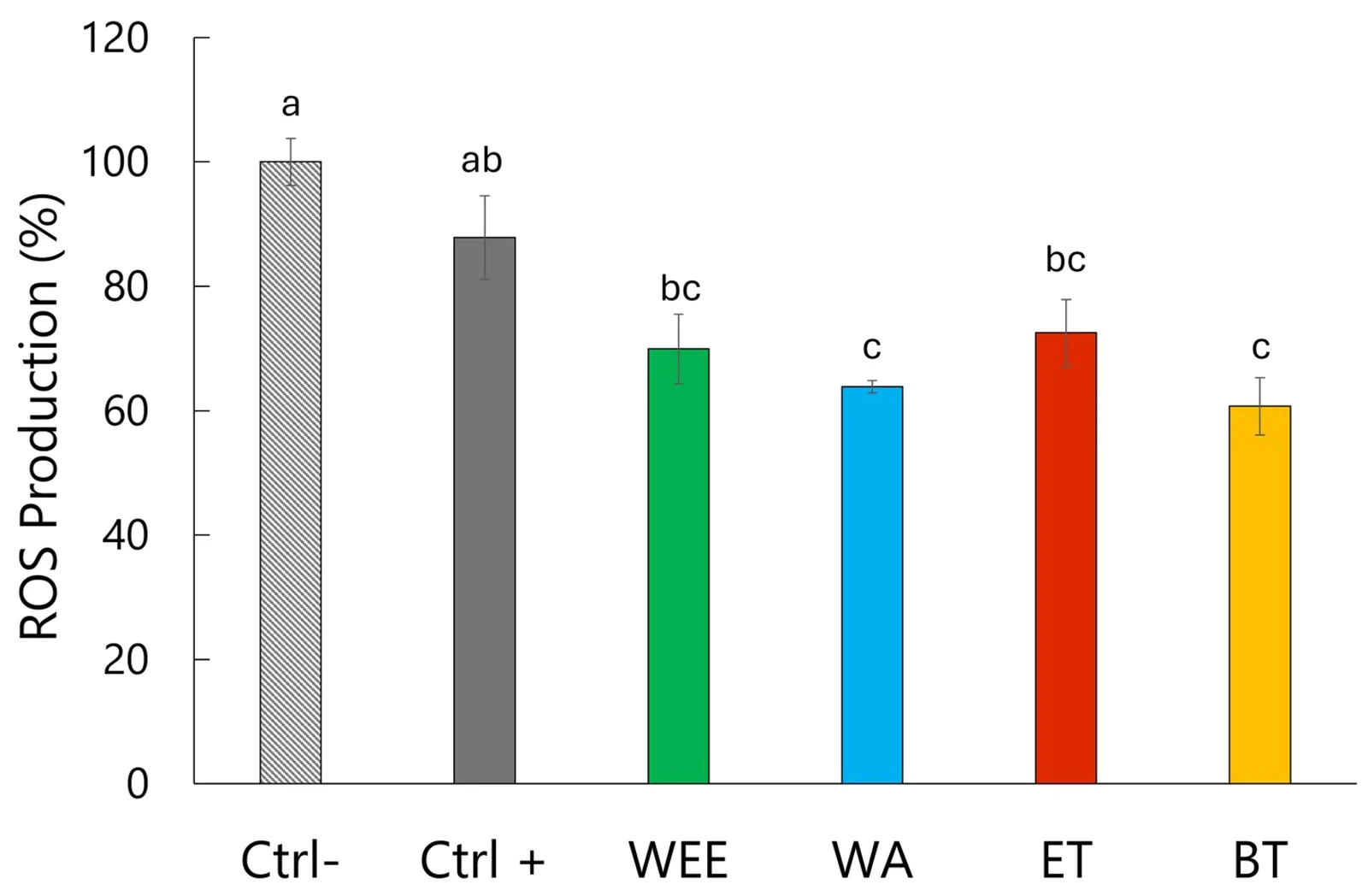
Effect of *Opuntia ficus-indica* (L.) (OFI) extracts on intracellular reactive oxygen species (ROS) production in an in vitro model of oleic acid (OA)-induced hepatic steatosis. Human hepatocellular (HepG2) cells were pre-treated with 300 µM OA to induce steatosis, then treated with 100 µg/mL each OFI extract for an additional 24 h. ROS production in response to an oxidant stimulus (2,2′-azobis(2-amidinopropane) dihydrochloride, 500 µM) was assessed. The results are presented as mean ± standard deviation of three different experiments. ^a–c^ Different letters indicate significant differences between treatments determined by ANOVA analysis followed by the Tukey test (*p* < 0.05). Ctrl−: non-treated cells without OA, Ctrl+: non-treated cells + OA, WEE: whole ethanolic extract + OA, WA: water + OA, ET: 80% ethanol + OA, BT: *n*-butanol + OA.

All OFI extracts reduced reactive oxygen species (ROS) production compared with non-steatotic cells (Ctrl−) (Figure 5). Cells treated with *n*-butanol (BT) and water (WA) extracts showed the greatest reductions in ROS production by 36.2% and 39.3%, respectively, compared to non-oleic acid-treated cells or Ctrl−. Compared to Ctrl+, BT and WA extracts reduced ROS production by 23.9% and 27.1%, respectively. These results agree with previous studies reporting high antioxidant activity in water or butanolic extracts of *Opuntia* spp. extracts, even higher than ethanolic or methanolic extractions. Aqueous extracts from *Opuntia ficus-indica* (L.) cladodes reported higher free radical scavenging activity (DPPH) than the 80% ethanol extract.

This superior antioxidant activity was attributed to the highest levels of apigenin, caffeic acid, vanillin, tannic acid, benzoic acid, and rutin compared with the 80% ethanolic extract [67]. Likewise, aqueous extracts from *Opuntia ficus-indica* (L.) contain antioxidant polysaccharides composed of rhamnose, arabinose, and glucose, which act as hydroxyl radical scavengers and effectively inhibit the formation of malondialdehyde in biological systems [68].

Antioxidant behavior of BTs has also been reported for *Opuntia ficus-indica* (L.) red fruits and was associated with its highest content of flavonoids compared to 80% methanolic extract, including quercetin-3-O-galactoside and isorhamnetin-3-O-rutinoside, which were not detected in 80% methanolic extract [69]. Other authors also reported that compounds such as taxifolin, isolated from the *n*-butanolic extract of *Opuntia ficus-indica* (L.) fruits by high-speed countercurrent chromatography (HSCCC), significantly reduced hepatic damage caused by alcoholic oxidative stress by preserving the activities of glutathione reductase and glutathione peroxidase (GSH-Px) enzymes [70]. On the other hand, the 80% ethanol (ET) extract and WEE reduced ROS production by 15.29% and 17.89%, respectively, but these differences were not significant compared to BT, WA, or Ctrl+. This effect was expected, as *Opuntia* spp. hydroalcoholic extractions are widely reported to recover antioxidant compounds, mainly phenolic acids and flavonoids.

### 3.6. Compound Identification and Quantification by UPLC-PDA-QDA

The most abundant phenolic compounds in the extracts were phenolic acids and flavonoids, as documented in *Opuntia* spp. [71,72]. Table 2 presents the identification of the most abundant compounds detected in WEE, BT, ET, and WA extracts obtained from *Opuntia ficus-indica* (L.). On the phenolic acid side, the compounds identified were piscidic (peaks 1 and 2) and eucomic (peak 4) acids, as well as derivatives of sinapic and ferulic acids, which are widely reported in the literature [73]. On the other hand, all detected flavonoids were isorhamnetin and kaempferol derivatives in their glycosylated forms, finding diglycosides such as isorhamnetin-glucosyl-pentoside (peak 12), isorhamnetin-glucosyl-rhamnoside (peak 13), isorhamnetin-acylated-glycoside (peak 14), and isorhamnetin-pentosyl-rhamnoside (peak 15), and triglycosides such as kaempferol rhamnosyl-rhamnosyl-hexoside (peak 8), isorhamnetin-glucosyl-rhamnosyl-rhamnoside (peak 9), and isorhamnetin rhamnosyl-rutinoside (peak 10). Almost all the most abundant flavonoids were derivatives of isorhamnetin, accounting for 6 of the seven identified flavonoids, in agreement with other authors who report similar profiles characteristic of *Opuntia ficus-indica* (L.) [74,75].

The analysis of the phenolic composition of *Opuntia ficus-indica* (L.) (OFI) extracts showed a similar profile across all extracts, but with significant differences in phenolic compound concentrations within each OFI extract (Table 3). In WEE, WA, and ET extracts, piscidic acid (peak 2) was significantly higher (*p* < 0.05) than in the BT. In contrast, in the BT, the most abundant phenolic compound was isorhamnetin-glucosyl-rhamnosyl-rhamnoside (peak 9), with the highest value among the extracts.

Concentrations of each compound were compared between all extracts, observing that piscidic acid (peak 1) and its isomer (peak 2) were more efficiently recovered in the WA extract, increasing their content by 1.82–1.83-fold in this extract in comparison with the direct extraction with 80% ethanol performed in WEE. In fact, phenolic acids represented 83% of the phenolic compounds quantified in the WA extract, whereas flavonoids accounted only for 17% (Figure S2). In contrast, the BT showed an inverse phenolic profile, with flavonoids accounting for 78% of all phenolic compounds quantified, while phenolic acids accounted for only 22%. In turn, among the flavonol glycosides identified and quantified in BTs, the triglycoside isorhamnetin-glucosyl-rhamnosyl-rhamnoside (peak 9) and its isomer (peak 10) represented almost 60% of all the flavonoids present in this extract, followed by diglycosides isorhamnetin-pentosyl-rhamnoside (peak 15) and isorhamnetin-glucosyl-rhamnoside (peak 13), representing 13.8% and 10.4%, respectively. Regarding the ET extract, its proportions of phenolic acids (50%) and flavonoids (50%) were very similar to those observed for the reference extract WEE, confirming the equivalence between the two extractions, regardless of the extraction strategy used. These findings suggest that the flavanol glycosides, isorhamnetin-glucosyl-rhamnosyl-rhamnoside (peaks 9 and 10), isorhamnetin-glucosyl-rhamnoside (peak 13), and isorhamnetin-pentosyl-rhamnoside (peak 15) may be closely related to the metabolic regulatory effects exerted by BTs on OA-induced steatotic cells. Furthermore, it was hypothesized that interactions between isorhamnetin flavanols and specific molecules, such as piscidic acids, may reduce the biological activity of flavanols. This hypothesis arose because, in the WEE used as a reference, which exhibited biological effects comparable to those of BTs, isorhamnetin-glucosyl-rhamnosyl-rhamnoside (peaks 9 and 10), isorhamnetin-glucosyl-rhamnoside (peak 13), and isorhamnetin-pentosyl-rhamnoside (peak 15) represented a statistically higher proportion (60%) in the extract than that represented by piscidic acids (40%), the most abundant phenolic acids in all extracts.

Some recent studies have shown that isorhamnetin has potential for treating MAFLD by modulating inflammation in MAFLD and MASH mouse models [76,77]. The isorhamnetin present in *Opuntia ficus-indica* (L.) is found mainly as glycosidic forms, which have the advantage of having a greater half-life than the isorhamnetin aglycone and also higher physiological stability during human-simulated digestion compared to its aglycone form [78]. The literature indicates that during flavanol glycoside absorption, these undergo deglycosylation in the small intestine by lactase-phlorizin hydrolase (LPH), releasing the flavanol aglycone or producing simpler glycosides, thereby exerting biological effects. Nevertheless, the specific flavanol structure affects this absorption process, which sometimes requires the sodium-dependent glucose transporter (SGLT1) to complete it [79,80]. The deglycosylation rate of flavonols depends on their structure and the position/nature of the sugar substitutions, where LPH more readily deglycosylates glucose sugar moieties. In contrast, rutinoside moieties are absorbed from the colon since they are not efficiently deglycosylated in the small intestine [81,82]. Also, associations with specific molecules can alter flavonol glycosides’ interactions with cells and, consequently, their biological activity.

#### Multivariate and Correlation Analysis

Principal components analysis (PCA) was performed to reduce dimensionality and identify associations between phytochemical profiles and the observed biological responses. The principal components, PC1 and PC2, accounted for 96.5% of the total variance (Figure S3). The PCA score plot shows that water (WA), ethanol (ET), and butanol (BT) extracts obtained from *Opuntia ficus-indica* (L.) by sequential exhaustive extraction (SEE) are distinct from one another. The WA extract showed positive loading on PC1, whereas the BTs showed positive loading on PC2, indicating differences in biological activity between the samples, driven by distinct dominant compound profiles (Table S2). The ET extract, located in the upper-right quadrant of the score plot (positive PC1 and PC2 values), was associated with high contributions from variables with positive loadings on both components, indicating the combined presence of phytochemicals determined as dominant in BT and WA extracts, and shares biological activities with them, but is not equal to either.

**Figure 6:**
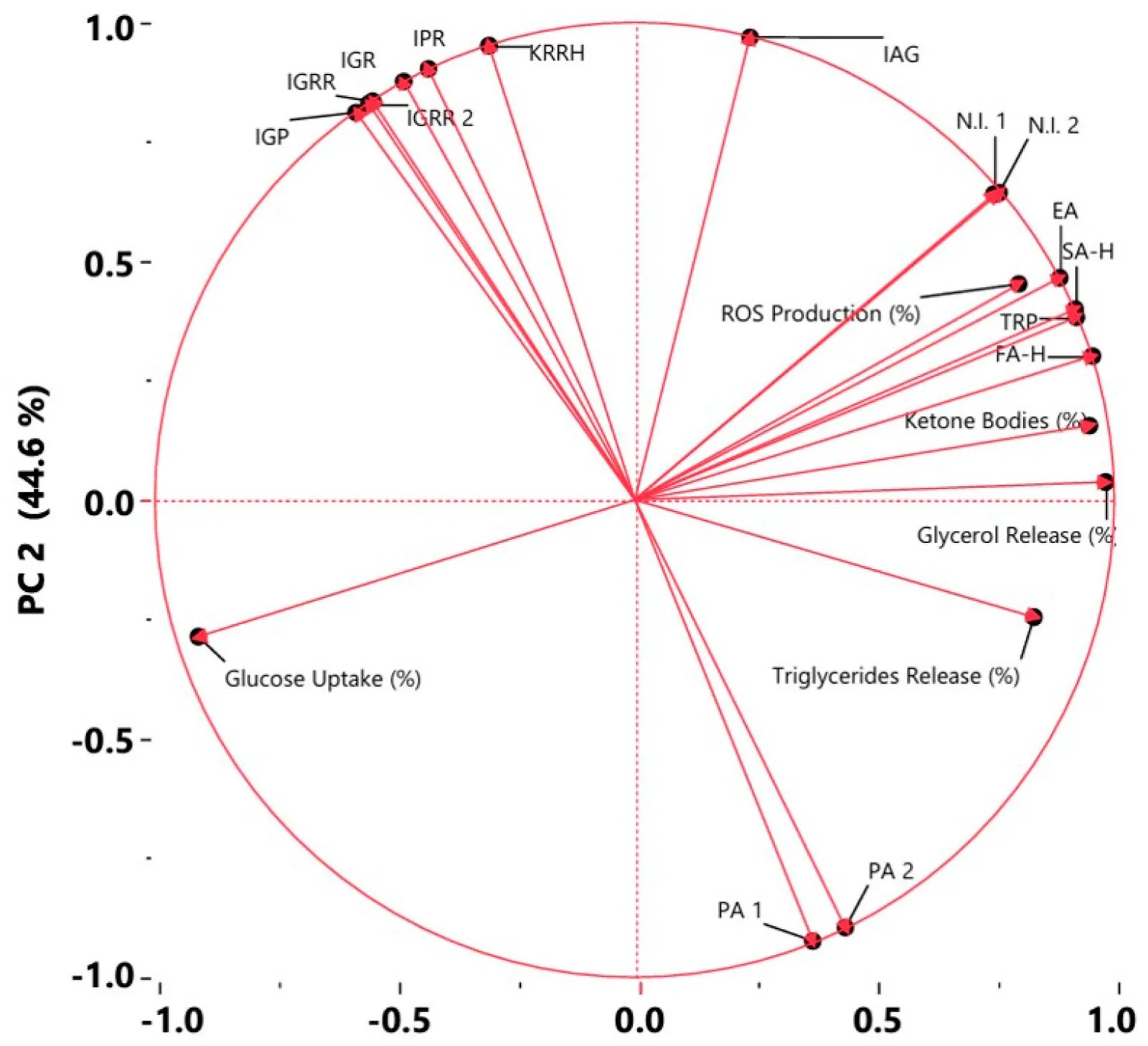
Loading plot from principal components analysis (PCA) integrating phenolic compound profile and biological responses of extracts obtained by sequential exhaustive extraction (SEE) from *Opuntia ficus-indica* (L.) (OFI) flour. Piscidic acid 1 (PA 1), Piscidic acid isomer (PA 2), tryptophan (TRP), eucomic acid (EA), sinapic acid-hexoside (SA-H), ferulic acid hexoside (FA-H), not identified 1 (N.I. 1), kaempferol rhamnosyl-rhamnosyl-hexoside (KRRH), isorhamnetin-glucosyl-rhamnosyl-rhamnoside (IGRR), isorhamnetin-glucosyl-rhamnosyl-rhamnoside isomer (IGRR 2), not identified 2 (N.I. 2), isorhamnetin-glucosyl-pentoside (IGP), isorhamnetin-glucosyl-rhamnoside (IGR), isorhamentin-acylated-glycoside (IAG), isorhamnetin-pentosyl-rhamnoside (IPR), principal component (PC).

The loading plot (Figure 6) and the variable contribution matrix (Table S2) indicate that PC1 is mainly driven by triglyceride and glycerol release, as well as by ketone body production, showing positive contributions from specific phenolic compounds such as eucomic acid (EA), sinapic acid-hexoside (SA-H), ferulic acid hexoside (FA-H), and the amino acid tryptophan (TRP). In contrast, isorhamnetin-glucosyl-rhamnosyl-rhamnoside (IGRR), isorhamnetin-glucosyl-rhamnosyl-rhamnoside isomer (IGRR 2), isorhamnetin-glucosyl-pentoside (IGP), and isorhamnetin-glucosyl-rhamnoside (IGR) showed a moderate negative loading on PC1. Likewise, kaempferol and isorhamnetin glycosides show positive loadings on PC2, whereas piscidic acids (PA 1 and PA 2) show negative loadings on PC2, suggesting an inverse relationship. Taken together, PCA showed that *Opuntia ficus-indica* (L.) extracts can differentially activate lipid metabolism-related pathways depending on their specific phytochemical profile: phenolic acids or glycosylated flavonoids. The evaluation of isolated compounds should be addressed to validate the activity association determined by the PCA.

**Figure 7:**
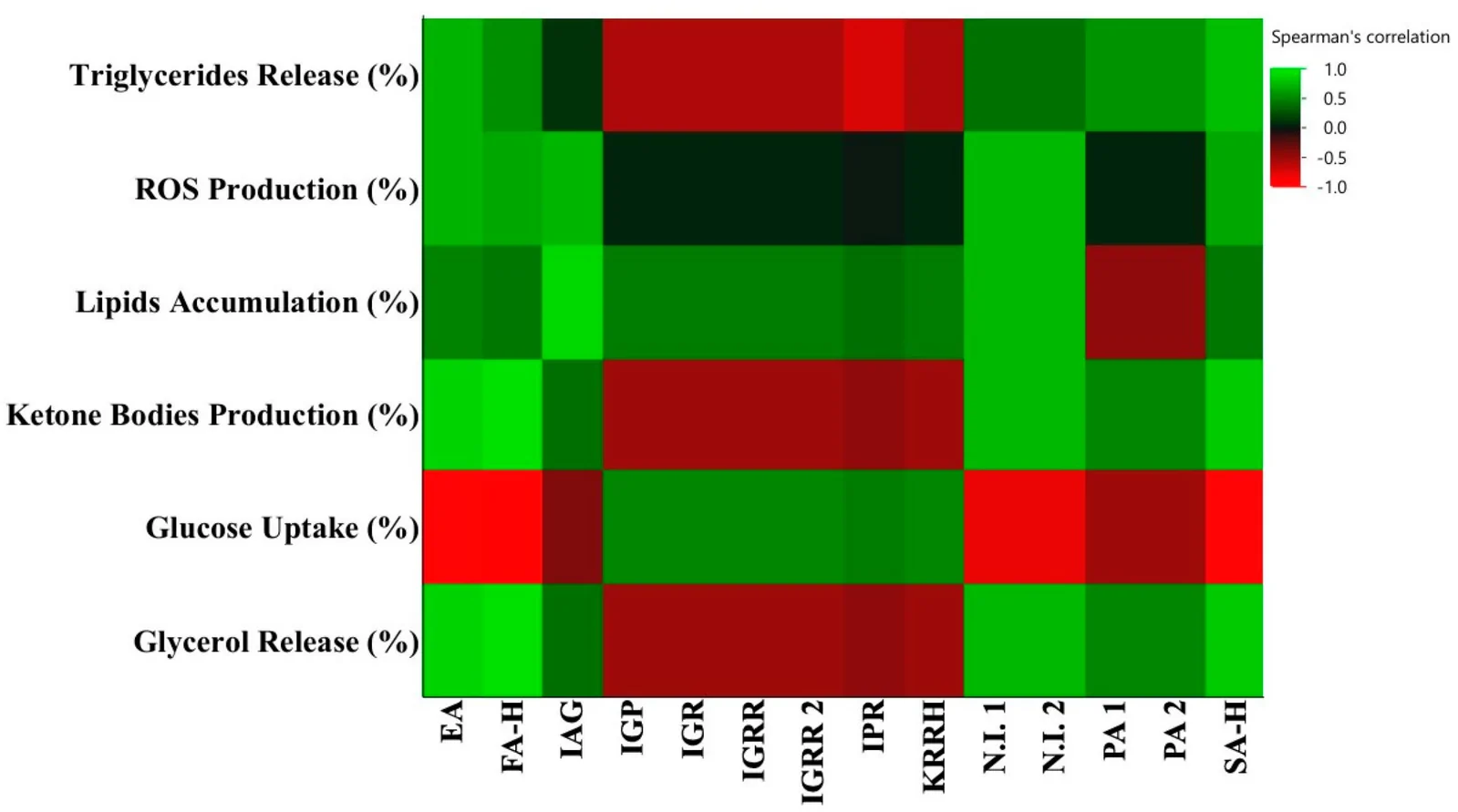
Spearman’s correlation heatmap shows associations between phenolic compounds and metabolic biomarkers of metabolic dysfunction and fatty liver disease (MAFLD). Piscidic acid 1 (PA 1), Piscidic acid isomer (PA 2), tryptophan (TRP), eucomic acid (EA), sinapic acid-hexoside (SA-H), ferulic acid hexoside (FA-H), not identified 1 (N.I. 1), kaempferol rhamnosyl-rhamnosyl-hexoside (KRRH), isorhamnetin-glucosyl-rhamnosyl-rhamnoside (IGRR), isorhamnetin-glucosyl-rhamnosyl-rhamnoside isomer (IGRR 2), not identified 2 (N.I. 2), isorhamnetin-glucosyl-pentoside (IGP), isorhamnetin-glucosyl-rhamnoside (IGR), isorhamentin-acylated-glycoside (IAG), isorhamnetin-pentosyl-rhamnoside (IPR), reactive oxygen species (ROS).

Spearman’s correlation analysis was performed between phenolic compounds and triglyceride release, glycerol release, ketone body release, glucose uptake, and ROS production (Figure 7). Ketone body production, triglyceride release, and glycerol release showed strong negative correlations with the content of isorhamnetin and kaempferol glycosides. Isorhamnetin-glucosyl-pentoside (IGP) and isorhamnetin-glucosyl-rhamnosyl-rhamnoside (IGRR) showed the strongest negative correlation with triglyceride release. In turn, phenolic acids, including eucomic acid (EA), sinapic acid-hexoside (SA-H), ferulic acid hexoside (FA-H), and piscidic acids (PA 1 and PA 2), showed a strong negative correlation with glucose uptake, whereas glycosylated flavonoids seem to improve this metabolic flux. Moderate negative correlations were observed between ROS production and specific compounds, including PA 1, PA 2, IFP, IGR, IGRR, IGRR2, IPR, and KRRH.

Overall, multivariate and correlation analyses suggest that OFI extracts are richest in glycosylated flavonoids, such as WEE and BTs, and potentially modulate triglyceride metabolism and lipolysis, enhance glucose metabolism, and reduce oxidative stress in steatotic hepatic cells, thereby maintaining energetic homeostasis and assuring their survival.

## 4. Conclusions

The treatment of MAFLD and other diet-associated chronic diseases requires incorporating new molecules or ingredients into disease management strategies, either as dietary components or as adjuncts to pharmacological therapy. In this context, *Opuntia ficus-indica* (L.) emerges as a promising source of bioactive compounds with antisteatotic, antioxidant, and metabolic reprogramming effects, making it a valuable candidate for the development of novel functional ingredients or a strategic ally in dietary interventions. In this study, isorhamnetin-glucosyl-rhamnosyl-rhamnoside, isorhamnetin-glucosyl-rhamnoside, and, to a lesser extent, isorhamnetin-pentosyl-rhamnoside were identified as the candidate compounds potentially driving these protective properties against MAFLD.

Nevertheless, these findings should be interpreted with caution, given methodological limitations of this study, including the use of a two-dimensional culture model based solely on the HepG2 cell line and the evaluation of OFI fractions at a single concentration. To address these limitations, future studies should confirm these effects using isolated, purified compounds and evaluate them in more physiologically relevant models, such as 3D cultures and in vivo animal models, alongside mechanistic assays. Addressing these gaps will be paramount to translating these initial results into practice. Ultimately, these findings underscore significant implications for designing personalized dietary strategies, opening new avenues to tailor ingredients or foods to an individual’s specific metabolic needs. Moreover, promoting such novel food sources not only improves nutritional status and health but also encourages the consumption of climate-resilient, sustainable crops.

## Figures and Tables

**Table 1:** Extraction yield of extracts obtained from *Opuntia ficus-indica* (L.) (OFI) flour using different solvents.

Extraction Method	Extraction Solvent	Dielectric Constant	Yield (mg/g of OFI Flour)
SEE	HX	1.89 ^a^	6.87 ± 1.34
	PE	2.00 ^b^	5.10 ± 1.36
	EA	6.02 ^a^	12.18 ± 0.60
	BT	17.80 ^a^	26.64 ± 1.82
	ET	33.90 ^c^	58.85 ± 0.16
	WA	80.00 ^a^	147.12 ± 13.66
	Total Extract Recovery		256.16 * ± 13.95
Hydroalcoholic extraction	WEE	50	99.86 ± 5.25

SEE: sequential exhaustive extraction; HX: *n*-hexane extract; PE: petroleum ether extract; EA: ethyl acetate extract; BT: *n*-butanol extract; ET: ethanol: water (80:20, *v*/*v*) extract; WA: water extract; WEE: whole ethanolic extract. * Significant difference between Total Extract Recovery and WEE yields determined by t-student test (*p* = 0.0006). ^a^ Information taken from [39] ^b^ Information taken from [36] ^c^ Information taken from [37,40,41,42].

**Table 2:** Identification and quantification of the most abundant phenolic compounds in *Opuntia ficus-indica* (L.) (OFI) extracts analyzed by ultra-performance liquid chromatography coupled with photodiode array and quadrupole mass detectors (UPLC-PDA-QDA).

Peak No.	Retention Time (min)	Compound Name	λ Max (nm)	*m*/*z* [M + H]^+^
1	3.71	Piscidic Acid	274	279 [M + Na]^+^
2	3.99	Piscidic Acid (Isomer)	274	279 [M + Na]^+^
3	7.05	Tryptophan	271	205
4	8.37	Eucomic acid	274	241
5	9.42	Sinapic acid-hexoside	286	415
6	10.08	Ferulic acid hexoside	330	379 [M + Na]^+^
7	10.59	N.I. 1	297	-
8	13.83	Kaempferol rhamnosyl-rhamnosyl-hexoside	264, 348	741
9	14.11	Isorhamnetin-glucosyl-rhamnosyl-rhamnoside	254, 354	771
10	14.35	Isorhamnetin rhamnosyl- rutinoside	254, 354	771
11	14.88	N.I. 2	288	-
12	15.39	Isorhamnetin-glucosyl-pentoside	254, 353	633 [M + Na]^+^
13	15.98	Isorhamnetin-glucosyl-rhamnoside	254, 354	625
14	16.51	Isorhamentin-acylated-glycoside	251, 331	769
15	17.21	Isorhamnetin -pentosyl-rhamnoside	254, 351	595

N.I.: Not Identified.

**Table 3:** Quantification of the most abundant phenolic compounds in *Opuntia ficus-indica* (L.) (OFI) extracts analyzed by ultra-performance liquid chromatography coupled with photodiode array and quadrupole mass detectors (UPLC-PDA-QDA).

Peak N°	Concentration (µg eq/g OFI Extract)			
	Hydroalcoholic Extraction	Sequential Exhaustive Extraction (SEE)		
	WEE	WA	ET	BT
1 ^¥^	2333.41 ± 77.40	4457.95 ± 129.87 ^a,^*	2012.88 ± 167.83 ^b^	1342.11 ± 175.11 ^c,^*
2 ^¥^	4510.35 ± 179.41	8695.97 ± 244.18 ^a,^*	4099.64 ± 405.72 ^b^	2265.20 ± 263.96 ^c,^*
3 ^¥^	1305.82 ± 59.49	272.58 ± 27.30 ^b,^*	910.99 ± 74.30 ^a,^*	–
4 ^¥^	1388.35 ± 100.36	251.71 ± 15.15 ^b,^*	1148.58 ± 23.56 ^a,^*	–
5 ^¥^	1565.58 ± 104.42	400.20 ± 31.49 ^b,^*	1375.91 ± 39.80 ^a^	–
6 ^ɣ^	142.31 ± 7.89	37.97 ± 2.93 ^b,^*	99.73 ± 3.21 ^a^*	–
7 ^¥^	182.18 ± 46.20	–	225.26 ± 55.97	–
8 ^§^	860.99 ± 27.32	296.35 ± 1.96 ^b,^*	702.96 ± 32.83 ^a,^*	775.26 ± 49.94 ^a^
9 ^§^	3991.43 ± 172.01	830.77 ± 53.36 ^c,^*	2890.76 ± 161.96 ^b,^*	4278.94 ± 342.82 ^a^
10 ^§^	3048.95 ± 134.99	630.92 ± 30.00 ^c,^*	2225.61 ± 155.58 ^b,^*	3338.88 ± 267.77 ^a^
11 ^¥^	512.65 ± 67.68	–	400.57 ± 63.35	–
12 ^§^	1014.80 ± 56.76	253.56 ± 28.60 ^c,^*	699.36 ± 62.91 ^b,^*	1049.11 ± 70.07 ^a^
13 ^§^	1344.28 ± 89.51	315.18 ± 27.53 ^c,^*	986.95 ± 71.54 ^b,^*	1322.06 ± 61.80 ^a^
14 ^ɣ^	387.33 ± 23.49	84.19 ± 6.82 ^c^*	276.60 ± 26.62 ^a,^*	198.34 ± 15.91 ^b,^*
15 ^§^	1898.42 ± 100.61	389.09 ± 16.69 ^c,^*	1375.99 ± 133.69 ^b,^*	1755.63 ± 22.70 ^a^
Total (mg/g OFI extract)	24.49 ± 0.07	16.92 ± 0.17 ^a^	19.43 ± 0.13 ^a^	16.33 ± 1.27 ^a^

WEE: whole ethanolic extract; WA: water extract; ET: 80% ethanol extract; BT: *n*-butanol extract. ^¥^ Concentration expressed as µg equivalents (eq) of gallic acid/g OFI extract. ^ɣ^ Concentration expressed as µg equivalents (eq) of ferulic acid/g OFI extract. ^§^ Concentration expressed as µg equivalents (eq) of quercetin/g OFI extract. Piscidic acid (1), piscidic acid isomer (2), tryptophan (3), eucomic acid (4), sinapic acid-hexoside (5), ferulic acid hexoside (6), not identified (7), kaempferol rhamnosyl-rhamnosyl-hexoside (8), isorhamnetin-glucosyl-rhamnosyl-rhamnoside (9), isorhamnetin rhamnosyl-rutinoside (10), not identified (11), isorhamnetin-glucosyl-pentoside (12), isorhamnetin-glucosyl-rhamnoside (13), isorhamentin-acylated-glycoside (14), isorhamnetin-pentosyl-rhamnoside (15). ^a–c^ Different lower-case letters indicate significant differences in each compound between different OFI extracts determined by ANOVA analysis followed by the Tukey test (*p* < 0.05). * Indicates significant differences in each compound between the OFI extracts and WEE determined by one-way ANOVA followed by Dunnett’s test (*p* < 0.05) using WEE as control.

## Data Availability

The original contributions presented in this study are included in the article/Supplementary Material. Further inquiries can be directed to the corresponding author.

## Supplementary Materials

The following supporting information can be downloaded at: https://www.mdpi.com/article/10.3390/foods15162939/s1, Table S1. Proximate composition of *Opuntia ficus-indica* (L.) cladodes flour; Figure S1. UPLC-PDA-QDA chromatograms of the whole ethanolic extract (WEE) and the WAs, ETs, and BTs obtained by sequential exhaustive extraction (SEE) from *Opuntia ficus-indica* (L.) (OFI) flour at 280 nm (A), 320 nm (B), and 365 nm (C); Figure S2. Composition of phenolic acids and flavonoids present in WEEs, WAs, ETs, and BTs obtained from *Opuntia ficus-indica* (L.); Figure S3. Score plot showing sample distribution analyzed by principal components integrating phenolic compounds profile and biological responses of extracts obtained by sequential exhaustive extraction (SEE) from *Opuntia ficus-indica* (L.) (OFI) flour; Table S2. Participation of each variable analyzed in the principal component analysis on each component.

## Abbreviations

The following abbreviations are used in this manuscript:

MAFLD	Metabolic dysfunction-Associated Fatty Liver Disease
MASH	Metabolic dysfunction-Associated Steatohepatitis
NAFLD	Non-Alcoholic Fatty Liver Disease
OFI	*Opuntia ficus inidica* (L.)
WEE	Whole Ethanolic Extract
SEE	Sequential Exhaustive Extraction
HX	*n*-Hexane
PE	Petroleum Ether
EA	Ethyl Acetate
BT	*n*-Butanol
ET	Ethanol
WA	Water
OA	Oleic Acid
MTT	3-(4,5-dimethylthiazol-2-yl)-2,5-diphenyltetrazolium bromide
DMSO	Dimethyl sulfoxide
ORO	Oil red staining
DCFH-DA	2′,7′-dichlorofluorescein diacetate
AAPH	2,2′-azobis(2-amidinopropane) dihydrochloride
ROS	Reactive Oxygen Species
NADPH	Nicotinamide adenine dinucleotide phosphate
UPLC	Ultra-High-Performance Liquid Chromatography
PDA	Photodiode-array
QDa	Quadrupole Dalton
N.I.	Not Identified
PA 1	Piscidic acid
PA 2	Piscidic acid isomer
TRP	Tryptophan
EA	Eucomic Acid
SA-H	Sinapic acid-hexoside
FA-H	Ferulic acid-hexoside
KRRH	Kaempferol rhamnosyl-rhamnosyl-hexoside
IGRR	Isorhamnetin-glucosyl-rhamnosyl-rhamnoside
IGRR2	Isorhamnetin-glucosyl-rhamnosyl-rhamnoside isomer
IGP	Isorhamnetin-glucosyl-pentoside
IGR	Isorhamnetin-glucosyl-rhamnoside
IAG	Isorhamentin-acylated-glycoside
IPR	Isorhamnetin-pentosyl-rhamnoside
PCA	Principal component analysis
