# Supplementary material for: Sequential Fractionation of Opuntia ficus-indica (L.) Reveals Isorhamnetin Glycosides Associated with Reduced Hepatic Lipid Accumulation and Oxidative Stress
Source: Foods. 2026 Aug 21;15(16):2939. doi: 10.3390/foods15162939 (PMC13512479; doi:10.3390/foods15162939)
Supplement: Supplementary file 1 [file foods-15-02939-s001.zip › foods-4452364-supplementary.pdf]

foods

MDPI

Supplementary

Sequential Fractionation of *Opuntia ficus-indica* (L.) Reveals Isorhamnetin Glycosides Associated with Reduced Hepatic Lipid Accumulation and Oxidative Stress

Jorge Alberto Uribe-Echeverría[1,2] and Marilena Antunes-Ricardo[1,2,] *

[1] School of Engineering and Science, Tecnologico de Monterrey, Ave. Eugenio Garza Sada 2501 Sur, Col: Tecnológico, Monterrey 64700, NL, Mexico
[2] Institute for Obesity Research, Tecnologico de Monterrey, Ave. Eugenio Garza Sada 2501 Sur, Col: Tecnológico, Monterrey 64700, NL, Mexico
* Correspondence: marilena.antunes@tec.mx

Table S1. Proximate composition of *Opuntia ficus-indica* (L.) cladodes flour

Component	Content (g/ 100 g)		
Lipids	3.18	±	0.15
Ashes	29.77	±	0.04
Protein	7.02	±	0.25
Soluble dietary fiber	12.09	±	0.90
Insoluble dietary fiber	32.72	±	0.37
Carbohydrates[1]	15.22	±	1.31

Values are expressed as mean ± standard deviation (SD) of three independent determinations (n = 3). Results are reported on a dry weight (DW) basis. 1. Available carbohydrates were calculated by difference.

https://doi.org/10.3390/foods15162939

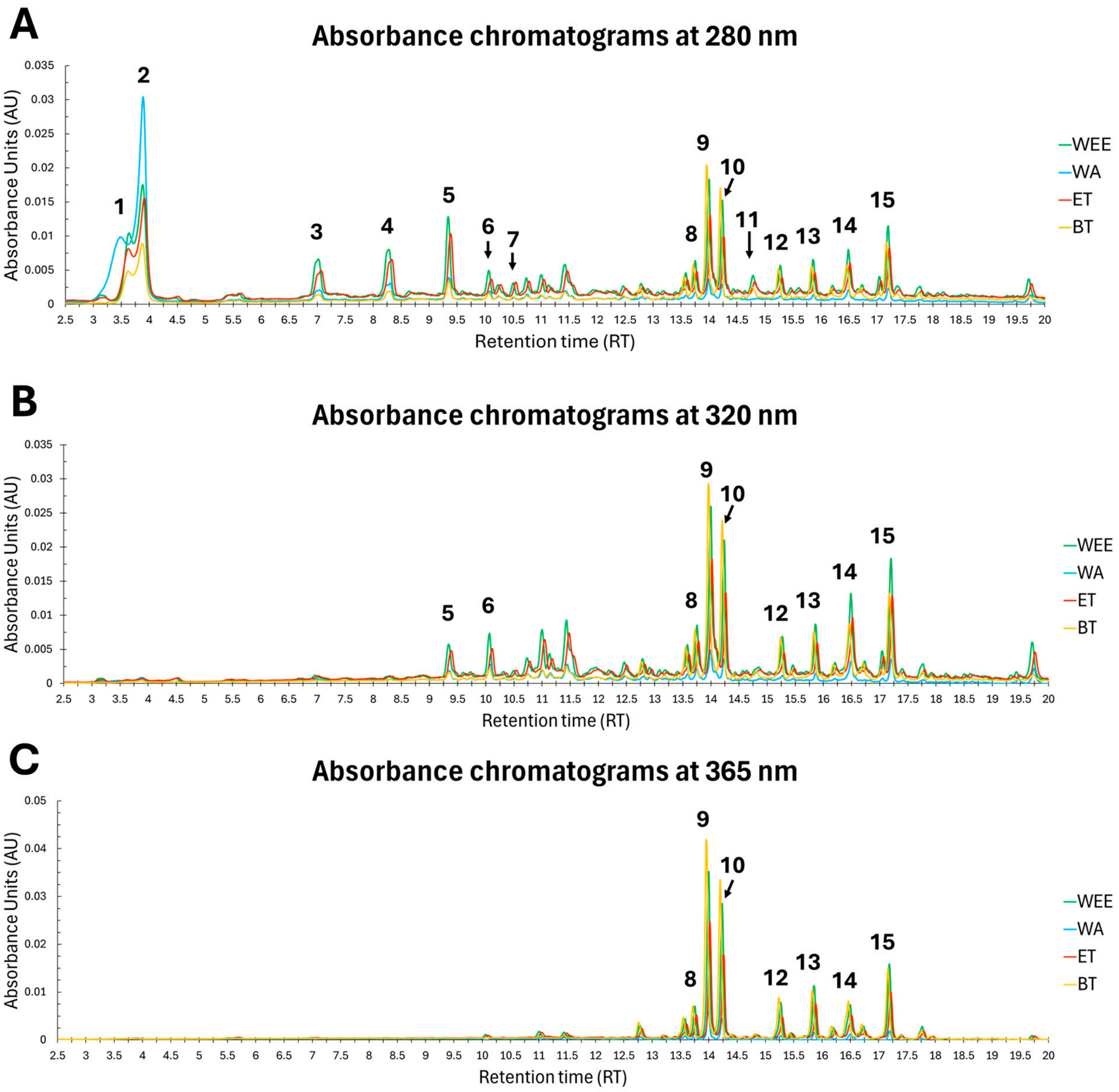

Figure S1. **UPLC-PDA-QDA chromatograms of the whole ethanolic extract (WEE) and the WA, ET, and BT extracts obtained by sequential exhaustive extraction (SEE) from *Opuntia ficus-indica* (L.) (OFI) flour at 280 nm (A), 320 nm (B), and 365 nm(C).** BT: butanol extract; ET: 80% ethanol extract; WA: water extract. **1**, Piscidic acid; **2**, Piscidic acid (isomer); **3**, Tryptophan; **4**, Eucomic acid; **5**, Sinapic acid hexoside; **6**, Ferulic acid hexoside; **7**, N.I. 1; **8**, Kaempferol rhamnosyl-rhamnosyl-hexoside; **9**, Isorhamnetin-glucosyl-rhamnosyl-rhamnoside; **10**, Isorhamnetin rhamnosyl-rutinoside; **11**, N.I. 2; **12**, Isorhamnetin-glucosyl-pentoside; **13**, Isorhamnetin-glucosyl-rhamnoside; **14**, Isorhamnetin acylated glycoside; **15**, Isorhamnetin-pentosyl-rhamnoside. N.I., Not Identified.

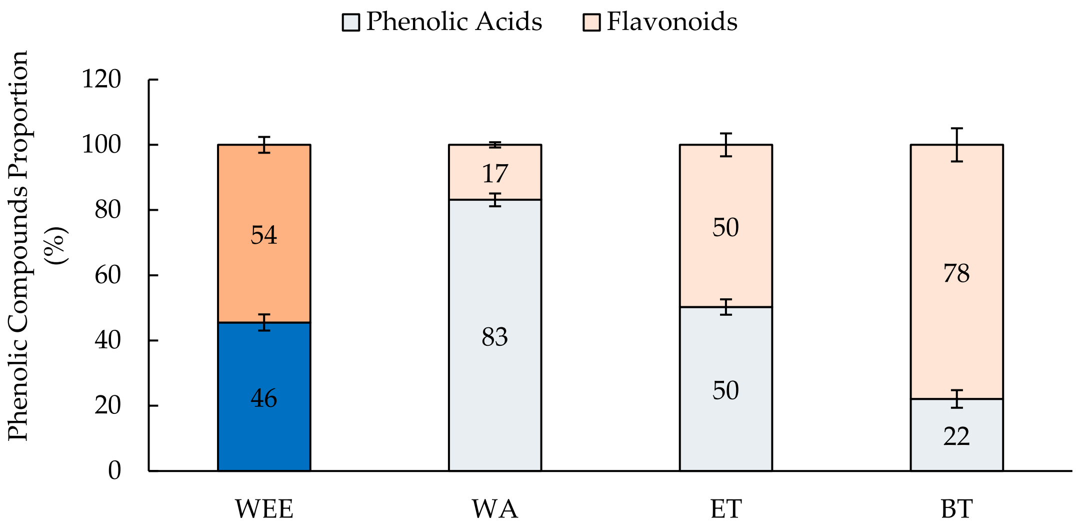

Figure S2. Proportion of phenolic acids and flavonoids present in WEE, WA, ET, and BT extracts obtained from *Opuntia ficus-indica* (L.). WEE: whole ethanolic extract obtained from *Opuntia ficus-indica* (L.) flour at a 1:10 (w/v) ratio and 80% ethanol: water (v/v). WA: water extract, ET: 80% ethanol extract, and BT: butanol extract obtained from *Opuntia ficus-indica* (L.) by sequential exhaustive extraction (SEE).

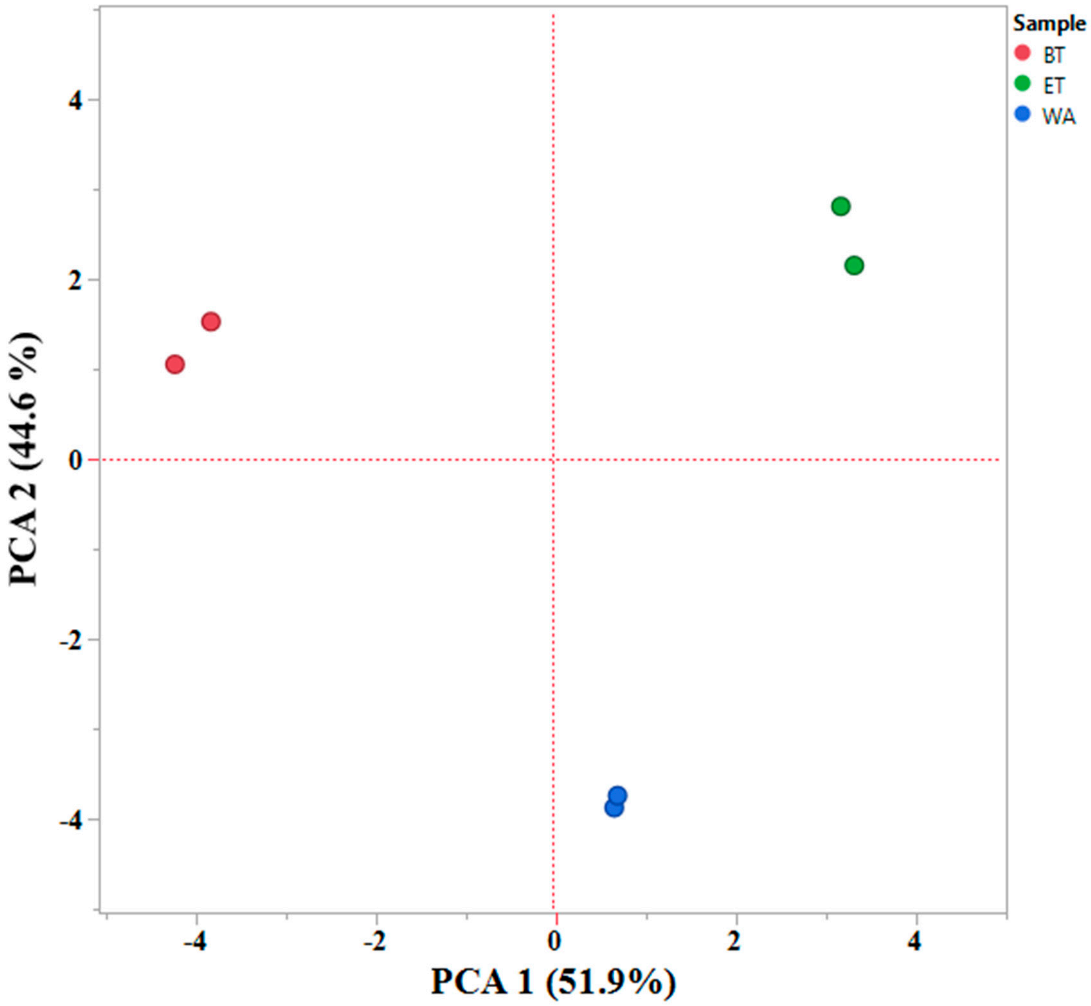

Figure S3. **Score plot showing sample distribution analyzed by principal components integrating phenolic compounds profile and biological responses of extracts obtained by sequential exhaustive extraction (SEE) from *Opuntia ficus-indica* (L.) (OFI) flour.** PCA: Principal component analysis; BT: butanol extract; ET: 80% ethanol extract; WA: water extract.

Table S2. Participation of each variable analyzed in the Principal Component Analysis on each component

Variable	PC1	PC2	PC3	PC4	PC5	PC6	PC7
Triglycerides Release (%)	0.83	-0.25	0.44	-0.21	0.10	-0.03	0.04
Glycerol Release (%)	0.98	0.04	-0.08	0.12	-0.02	0.12	0.08
ROS Production (%)	0.80	0.45	0.34	0.20	-0.15	0.00	-0.02
Glucose Uptake (%)	-0.91	-0.29	0.26	0.20	0.10	0.03	-0.06
Ketone Bodies (%)	0.95	0.16	-0.13	0.15	0.24	-0.03	-0.01
PA 1	0.37	-0.92	0.06	0.04	0.04	0.04	0.03
PA 2	0.44	-0.90	0.05	0.04	0.03	0.03	0.03
TRP	0.92	0.38	-0.05	0.06	-0.03	-0.10	0.00
EA	0.89	0.46	-0.04	0.02	-0.02	-0.05	-0.01
SA-H	0.92	0.40	-0.02	-0.01	-0.01	-0.03	-0.02
FA-H	0.95	0.30	-0.06	0.04	-0.02	-0.02	0.01
N.I. 1	0.75	0.64	0.01	-0.12	0.02	0.10	-0.06
KRRH	-0.30	0.95	0.03	0.03	0.03	0.00	0.03
IGRR	-0.55	0.83	0.05	0.04	0.03	-0.01	0.04
IGRR 2	-0.55	0.83	0.05	0.03	0.03	0.00	0.04
N.I. 2	0.76	0.64	-0.01	-0.07	0.01	0.05	-0.04
IGP	-0.58	0.81	0.06	0.00	0.04	0.00	0.02
IGR	-0.48	0.88	0.03	0.00	0.02	-0.01	0.01
IAG	0.24	0.97	0.05	-0.03	0.04	0.02	-0.01
IPR	-0.43	0.90	-0.02	-0.02	0.00	0.04	0.00

PA 1: piscidic acid 1; PA 2: piscidic acid isomer, TRP: tryptophan; EA: eucomic acid; SA-H sinapic acid-hexoside; FA-H: ferulic acid hexoside; N.I.: not identified; KRRH: kaempferol rhamnosyl-rhamnosyl-hexoside, IGRR: isorhamnetin-glucosyl-rhamnosyl-rhamnoside, IGRR 2: isorhamnetin-glucosyl-rhamnosyl-rhamnoside isomer; IGP: isorhamnetin-glucosyl-pentoside; IGR: isorhamnetin-glucosyl-rhamnoside; IAH:isorhamentin-acylated-glycoside; IPR: isorhamnetin-pentosyl-rhamnoside; ROS: reactive oxygen species; PC: principal components.

Disclaimer/Publisher's Note: The statements, opinions and data contained in all publications are solely those of the individual author(s) and contributor(s) and not of MDPI and/or the editor(s). MDPI and/or the editor(s) disclaim responsibility for any injury to people or property resulting from any ideas, methods, instructions or products referred to in the content.

https://doi.org/10.3390/foods15162939
